# The role of learner engagement with corrective feedback in EFL/ESL classrooms

**DOI:** 10.3389/fpsyg.2023.1118467

**Published:** 2023-01-24

**Authors:** Hao Liu, Mengya Feng

**Affiliations:** Department of English, School of Languages and Culture, Tianjin University of Technology, Tianjin, China

**Keywords:** learner engagement, corrective feedback, EFL/ESL, engagement training, L2 learning

## Abstract

As corrective feedback (CF) is conducive to students’ second language (L2) development, a considerable number of studies have investigated the effects of different types of CF strategies on EFL/ESL learning achievement. However, the role of learner engagement has been largely neglected in the field of CF research. The present study aims to describe the role of learner engagement with CF in EFL/ESL classrooms by reviewing theoretical and empirical evidence. The findings reveal that learner engagement is indispensable for CF to be effective, and providing strategies for working with CF is essential to guide EFL/ESL learners in their learning process. The implications for teacher CF practice and learner engagement training are also discussed.

## Introduction

Corrective feedback (CF) has always been an important part of EFL/ESL classrooms (Hyland and Hyland, 2019). A substantial number of studies have been conducted to identify categories of CF strategies and to investigate the effects of different types of CF strategies on second language (L2) development (Lee and Lyster, 2016; Sato and Loewen, 2018; Fu and Li, 2022). The reason for the intense interest in CF lies partly in the theoretical significance of testing the claims of various competing second language acquisition (SLA) theories (e.g., cognitive theory, skill-learning theory, and sociocultural theory), which see different roles for CF in promoting learners’ knowledge of their L2. Moreover, CF is a highly researchable phenomenon that can be identified and manipulated with relative ease (Ellis, 2010). Additionally, CF research is practically relevant to L2 teaching, providing implications for improving pedagogy to assist L2 students’ acquisition of the target language and help them improve the effectiveness of their learning (Hiver et al., 2020).

However, CF alone is not enough to directly bring about acquisition. Feedback is “a long-term dialogic process in which all parties are engaged” (Price et al., 2011, p. 879). In this vein, the effectiveness of feedback correlates significantly with how learners deal with CF during the learning process (Handley et al., 2011; Hiver et al., 2020). Accordingly, it is crucial to conceptualize L2 learners’ responsibility with CF and to motivate their proactive receptivity to feedback (Zhang, 2021). Only by meeting this criterion can learners be actively engaged with the CF provided and eventually benefit from it (Moser, 2020). As Wiliam (2011, p. 129) pointed out, “feedback should be more work for the recipient than the doner.” Ellis (2010) emphasized that learner engagement is an important factor, and there is a need to understand why and how L2 learners engage with CF. He delineated three aspects of learner engagement: cognitive, behavioral, and affective. Price et al. (2011) highlighted that these aspects are significant facets that impact students’ perceptions of teacher feedback. Shen and Chong (2022) acknowledged that researching learners’ reasons for and responses and reactions to working with feedback is beneficial to provide flexible feedback methods personalized for L2 learners’ needs.

Despite the importance of learner engagement in investigating CF, few empirical studies (Zheng and Yu, 2018; Vattøy and Smith, 2019; Shen and Chong, 2022) have explored the role of learner engagement with CF and its positive effect on students’ EFL/ESL performance. Furthermore, a detailed theoretical description of the association between these variables, and in particular, the beneficial consequences for L2 development, is lacking. Against this backdrop, the present review study aims to explicate the conceptualization of CF and learner engagement, their interrelationships, and the positive effect on EFL/ESL learning in practical classrooms.

### Corrective feedback

CF is theoretically assumed to facilitate the acquisition of L2, especially when language learning primarily focuses on the linguistic form and its meaning (Sheen and Ellis, 2011). CF is generally conceptualized as “comments or other information that learners receive concerning their success on learning tasks or tests, either from the teacher or other persons” (Richards and Schmidt, 2002, p. 199). Drawing on Lyster and Ranta’s (1997) categorization and related studies, Sheen and Ellis (2011) described oral and written CF in more detail. For oral teacher feedback, they distinguished between input-providing feedback and output-prompting feedback and between implicit feedback and explicit feedback. The specific feedback strategies identified included explicit correction, recast, repetition, elicitation, metalinguistic clues, and clarification requests. In the case of written *CF* studies, the authors also categorized written teacher feedback into direct and indirect feedback, and metalinguistic information and no metalinguistic information, and created a table. Because of the importance of feedback in reducing discrepancies between L2 learners’ current understanding and expected performance, CF can be rewarding, with significant contributions to facilitating L2 learning (Han, 2019; Li and Vuono, 2019; Hiver et al., 2020). Research evidence shows that CF is a crucial element not only in providing feedback to L2 learners regarding the progress of their EFL/ESL learning but also in providing feed-forward for EFL/ESL learning outcomes (Lee and Lyster, 2016; Sato and Loewen, 2018; Fu and Li, 2022).

### Learner engagement

The notion of learner engagement generally refers to “the time and effort students devote to activities that are empirically linked to desired outcomes” (Kuh, 2009, p. 683). In this respect, learner engagement should consider students’ sense of self and their aspirations and contexts (Winstone and Carless, 2020). According to Han and Hyland (2015), learner engagement is a critical element that links the provision of CF and L2 learning outcomes. As this is a complex and multidimensional construct, three dimensions have been proposed for learner engagement: cognitive, affective, and behavioral (Eccles, 2016). Cognitive engagement concerns students’ mental efforts, mental activity, and complicated learning strategies employed to respond to the CF they receive. Affective engagement refers to students’ internal states and attitudinal responses or affective reactions to the CF. Behavioral engagement is related to the ways in which students become involved in developing their interlanguages (e.g., correcting errors, reformulating their oral discourse, and revising their written text). Research has revealed that learner engagement is a dynamic construct that evolves over time (Mercer, 2019). The levels of engagement in all three dimensions can fluctuate, and students cannot be engaged in all three dimensions with the same intensity at all times. Shen and Chong (2022) claimed that learner engagement is closely linked to learning outcomes, with different degrees of engagement predicting different responses to CF and different academic outcomes. Moser (2020) suggested that engaging students is not simply to promote their engaged time or attendance for academic performance. It requires a holistic understanding of students’ feelings, attitudes, and active behavior, as well as their psychological connections within academic contexts. These findings are helpful in conceptualizing the multidimensional nature of learner engagement, which can help improve learners’ educational experiences and academic performance.

### The role of learner engagement with corrective feedback

The positive role of learner engagement with CF can be illustrated through what Ellis (2010) articulated in a componential framework for investigating CF. He postulated that CF must be heeded if it is to have any effect on learners’ L2 development. Rather than blindly receiving feedback, L2 learners act as active agents who filter the CF, and their true involvement and meaningful participation are the only guarantee that learning will take place (Best et al., 2015; Enel and Yao, 2021). In this regard, learner engagement with CF is central to ensure learning outcomes, which mediate the efficiency and effectiveness of CF (Pitt and Norton, 2017). Moser (2020) posited that the three taxonomies of cognitive, affective, and behavioral engagement are at the heart of learners’ dynamic engagement processes. Zheng and Yu (2018) further clarified that the three dimensions are interrelated under the meta-construct of learner engagement with CF. Specifically, cognitive engagement refers to students’ cognitive investment and meta-cognitive operations in processing CF. It might vary from noticing or understanding the CF to revising to monitoring and regulating the mental effort of processing CF (Storch and Wigglesworth, 2010). At the affective level, learner engagement is manifested through L2 students’ attitudinal responses to the feedback they receive, including expressing emotions and feelings, showing personal and moral judgment, and appreciating the value of CF (Martin and Rose, 2002). Behaviorally, learner engagement with CF focuses on what students do with CF, and it might range from doing the assigned tasks to using strategies to improve their language performance, as required by the CF received from the teacher (Han, 2017).

To illustrate the importance of individual and contextual factors that mediate CF, Ellis (2010) asserted that these factors influence teachers’ provision of CF and learners’ responses to this CF. Additionally, as stated by Guilloteaux (2016), phenomenological, demographic, and instructional factors affect student engagement. In a similar vein, Moser (2020) proposed the engagement-mediator-feedback model and stated that students’ emotions, cognition, and behavior influence engagement, which then impacts L2 learning and achievement. The model also emphasized that the mediating factors impeding or fostering engagement should not be underestimated, and that these factors, including attitudes, emotions, peers, teachers, friends, and family, play a crucial role in maintaining and improving learner engagement with CF. Students who are intrinsically and extrinsically motivated are more engaged with the chosen feedback strategies in learning activities. According to Shen and Chong (2022), students’ engagement with CF is jointly mediated by learner factors, the micro-classroom context, and the macro-educational context. These studies illustrate the potential of understanding the complex and multifaceted nature of learner engagement with teacher CF and provide insight into how to explore the interrelationship between learner engagement and CF.

## Empirical studies

Due to the importance of learner engagement in investigating CF, several studies have recently been conducted to probe the interrelationship between learner engagement and CF, but limited attention has been paid to the relevance of learner engagement with CF for L2 learning and achievement. Empirical research using a dynamic and multifaceted perspective to examine the role of learner engagement with CF in EFL/ESL classrooms is still in its infancy. Zheng and Yu (2018), for instance, investigated lower-proficiency students’ engagement with written CF in EFL writing classes. Underpinned by a multidimensional conceptual framework and drawing data from multiple sources, the study found that students’ low proficiency may have a negative influence on learners’ cognitive and behavioral engagement with CF, leading to an imbalance in engagement across the three dimensions. Han and Hyland (2015) used a qualitative inquiry to examine four college students’ engagement with CF and reported that CF affects learner engagement and that students seem to process CF at the surface level. Their study also revealed that individual differences in learner engagement could be generated from learners’ beliefs, learning expectations, and the interactional contexts of receiving and processing CF. Mahfoodh (2017) examined the role of students’ affective engagement with CF in their text revisions and revealed that students’ perceptions and uptake of CF could be mediated by their affective and emotional responses, such as contentment and dissatisfaction. In a small-scale study, Moser (2020) examined the mediating factors impacting learners’ engagement with written CF and emphasized that a feedback-rich environment is necessary to support engagement with feedback. Similarly, formative assessment and a learning-oriented atmosphere should be created to foster learner engagement. Clear instructions and strategies for working with feedback should also be provided to facilitate learner engagement with CF, both written and oral. Vattøy and Smith (2019) examined students’ perceptions of teachers’ corrective feedback practice in the EFL teaching context. The Responsive Pedagogy Questionnaire was employed to explore students’ perceived external goal orientation, self-regulation, and self-efficacy. Based on multiple regression and path analyzes, the study revealed that students’ perceived self-efficacy could positively influence their engagement with teachers’ feedback. In a similar vein, Zhang and Hyland (2018) approached students’ engagement with teacher-written corrective feedback on second language writing and found that learners’ beliefs, language proficiency, and other individual factors could affect their affective, cognitive, and behavioral engagement with teacher feedback.

## Conclusion and pedagogical implications

This review article characterized the role of learner engagement with CF in EFL/ESL classrooms based on theoretical and empirical evidence. It can be concluded that learners’ engagement with CF can make a noticeable difference in EFL/ESL students’ perceptions, reactions, and responses to the feedback they receive, which will impact their L2 achievement in target-like form-meaning mappings. The theoretical and empirical evidence shows that learner engagement is a dynamic process that is likely to change throughout a learner’s education. The findings seem to be illuminative for L2 teachers to fine-tune their choice of corrective strategy to the developmental level of EFL/ESL learners. An important implication is that teachers should support students’ agenetic engagement with CF to motivate them to become more involved in the feedback and learning process. L2 learners are proactive recipients, and their engagement with, and use of, the feedback they receive is a critical determinant of feedback effectiveness (Winstone et al., 2016). Another important implication is that for CF to be effective, it is necessary to explain the feedback method in detail to learners (Carless and Winstone, 2023). Providing feedback for L2 learners remains one of the major tasks of language teachers everywhere. However, it is difficult for L2 learners to automatically know how to respond to the feedback they receive. Accordingly, the first step should be to engage students and emphasize their responsibilities. Therefore, teachers should provide specific strategy training to help students learn how to work with the feedback so that they can positively engage with it. Moreover, because teacher CF can positively influence students’ academic performance, EFL/ESL teachers who intend to promote students’ engagement with CF could enhance their instructional abilities. They should provide consistent, predictable, and contingent feedback and adjust their feedback strategies to the level of the L2 learners. Furthermore, teachers can communicate their expectations for providing feedback, which will help L2 learners understand the feedback and maximize their role in the feedback process.

## Author contributions

HL wrote the manuscript. MF drafted it. All authors contributed to the article and approved the submitted version.

## Funding

This paper was funded by the Project titled Optimization and Practice of Doing-Learning-Using Integrated EFL Platform Course supported by Tianjin Philosophy and Social Science Foundation (Grant Number: TJYY21-012).

## Conflict of interest

The authors declare that the research was conducted in the absence of any commercial or financial relationships that could be construed as a potential conflict of interest.

## Publisher’s note

All claims expressed in this article are solely those of the authors and do not necessarily represent those of their affiliated organizations, or those of the publisher, the editors and the reviewers. Any product that may be evaluated in this article, or claim that may be made by its manufacturer, is not guaranteed or endorsed by the publisher.
